# The Treatment of Summer Complaints by the Bromide of Potassium

**Published:** 1869-08

**Authors:** Salvatore Caro


### THE TREATMENT OF SUMMER COMPLAINTS BY THE BROMIDE OF POTASSIUM.

REMARKS MADE BEFORE THE MEDICAL SOCIETY OF THE COUNTY OF NEW YORK, JUNE 7TH, 1869.

BY SALVATORE CARO, M.D.

"Nihil est in intellectu quam prius fuerit in sensu."—ARISTOTLE.

MR. PRESIDENT and Fellow-Members of the Medical Society of the County of New York.

The paper which I am about to read before you and submit to your sound judgment, is not intended to destroy theories which have been adopted for a number of centuries by the profession generally, nor to create new ones, but simply to present you with facts that to me appear of great service to science and to humanity at large; particularly to those helpless beings concerning whom the question was lately raised, by a member of this distinguished body, as to the causes of their mortality. I have entitled this paper *The Treatment of Summer Complaints by the Bromide of Potassium.* Possibly the proper name would have been *Cholera Infantum;* but since adults as well as infants are sometimes troubled with these complaints, and have been cured by the same remedy, I concluded to adopt the former title.

Before entering the field of practical observations, permit me briefly to describe the causes, symptoms, and end of such a complaint.

*Causes.*—Heat, cold, and dampness, separately or combined, have a great tendency to produce diarrhœa and dysentery. Low, damp ground, badly ventilated and crowded apartments are sure to create this disorder. In the summer season it is supposed to be epidemic, coming in the other seasons only in a sporadic form. I have invariably found that the exposure of babies to night air, when affected with bowel complaint, aggravates the symptoms. The bathing of infants, either by sponging or immersion, whilst in a state of perspiration, is one of the most terrible causes of this affection. Atmospheric heat is the first cause of summer complaint. Its action upon the human body is peculiar, particularly in children, the external surface of the body being generally cooler, and the internal parts warmer under its influence.

Its first effects are to produce cutaneous perspiration, and to render more active the hepatic function, as has been clearly demonstrated by Dr. Jas. Johnson (*On Tropical Climates.* "The sympathy between skin and liver, under varying conditions of external warmth.") Whatever may be the explanation of the facts, we know by experience that high atmospheric heat, long continued, has a marked action upon the liver, increasing and altering the quality of the bile, not unfrequently producing inflammation of itself, and, through its protracted continuance, affecting the bowels and giving rise to vomiting and diarrhœa.

The skin and liver are not the only organs affected by heat. From the imperfect consolidation of the cranium, the sun's rays readily affect the brain, suddenly and extensively influencing the nervous system. To cite a familiar instance of this, in summer days we frequently see in the street or in the horse cars, a healthy baby exposed to the sun with uncovered head, commence vomiting, without any cause referable to the stomach. Until the sixth or seventh year, the brain being but imperfectly developed, the spinal system predominates over the cerebral, and this accounts for the predisposition to reflex action upon the stomach and digestive organs. When either the brain or spinal system is affected, exhaustion undermines the feeble vital powers of the child, producing a general relaxation of the intestines, attended with vomiting, diarrhœa, and hydrocephaloid symptoms.

The infant requires proportionately more food than the adult, having not only to repair the daily waste but also to provide for growth; and yet its digestive organs are weaker. To this we may attribute the constant disturbance of the child's stomach and bowels, particularly when fed with unhealthy food. To prevent this disorder the child should be nourished from the mother's breast; or, if deprived of this, by cow's milk, until the stomach has altered in form, gained greater muscularity, and, together with the other digestive organs, the power to assimilate food of a different nature. Until the appearance of the first molar tooth, the child should have no solid food, but be made to depend upon the mother's breast or its substitute.

Nausea and vomiting are the most common symptoms of indigestion during infancy. The vomiting of indigestion must be distinguished from the regurgitation which occurs without nausea, simply from an overloaded state of the stomach, where the mother has a great flow of milk, and the child partakes too freely. This regurgitation is not to be taken as a symptom of indigestion, but many times it is the cause of great disturbance to the child's digestive functions, by the frequent loading and unloading of the stomach; it should therefore be prevented. If what the child vomits after nursing is coagulated, with sour smell, indigestion is the cause. It is accompanied by griping pains, and immediately afterwards by sour and scanty evacuations. The coagulated milk, unless either digested or vomited, will be the cause of great pains, gastric inflammation, and diarrhœa.

At all ages, children that are weaned are more subject to indigestion, especially when fed with stimulating and indigestible food. The symptoms are then more severe. Vomiting of undigested food, with alvine discharges of a similar nature, are more frequent, and colic pains more violent. The child's appetite is increased; he eats with avidity and in great quantity; but the food is past almost unaltered, affording no nourishment to the system. He grows fretful, the brain becomes disturbed, convulsions often appearing; finally he dies of anæmia. If the cramming of the stomach is the only cause of the indigestion, and consequent bowel complaint, we should first remove the causa morbi, either by an emetic or an antacid, and then regulate the diet; under this simple treatment the child will recover. But, if the presence of the undigested food has produced a certain degree of disturbance in the hypogastric and solar plexuses and large semilunar ganglia, with their nervous supply to the vessels and viscera—the fons et origo of the disease known as cholera—neither emetics, antacids, nor opiates will relieve the sufferer as efficaciously as the bromide of potassium.

There is a still more formidable cause of summer complaint—dentition—which begins on an average, from the sixth to the eight month, terminating at the twenty-fourth or thirtieth (that is, for the deciduous teeth). There is no writer on the diseases of children that does not assert facts, more or less positive, to the effect that teething is the cause of summer complaint. I have invariably found it accompanied by vomiting and loose bowels; and in the majority of cases, I have observed these symptoms at the irruption of each tooth. It is not only the irritating influence of dentition that induces a peristaltic action of the bowels; we must not lose sight of the fact, that the digestive canal is simultaneously undergoing a certain evolution, in order to prepare it to receive food suitable for the maintenance of the body at this stage of its development. At the same time, the salivary glands commence to secrete saliva in abundance; and the whole glandular system assumes a rapid growth, and with it receives a new excitability, aggravating the exquisite sensitiveness of the growing parts, producing vomiting and diarrhœa. Admitting, however, that the disturbances arising at the time of dentition are but temporary, and chiefly of a nervous character, and finding the common every-day treatment fail of giving prompt relief, why do we not use a sedative to this over-excited system? In the most severe cases of odontitis, either with or without ulcerated gums or loose bowels, I have never failed to relieve the child by the local application of the bromide of potassium. Almost immediately after the first rubbing on the gums, from being turgid, swollen, and red, they assume their natural color, and a certain amount of ease is felt. Saliva commences to dribble; and, as if by enchantment, agitation, carpopedal involuntary motion, vomiting, and looseness of the bowels disappear. As the vomiting and diarrhœa in this case are not the consequence of gastro-enteritis, but of an excitement of the stomach and the intestinal mucous membrane, owing to the inflamed condition of the gums, I suppose it will never be cured either by the scarification of the gums, or by the use of astringents or anodynes; but, as I shall hereafter prove, simply by the use of the bromide of potassium. By microscopical examination I have found in the vomited matter only undigested food, in different stages of disintegration, and small particles of the mucous membrane of the stomach. The fæces in the beginning of the affection are like broken-down and half-digested particles of food. When the stools have turned green, they contain decomposed blood and bile; when white, large quantities of mucous membrane, and even matter; and, when red, blood. I have had no opportunity of making any anatomical sections, therefore can say nothing regarding the pathological condition of the internal organs; but I would submit it to our anatomists and microscopists to look into the trisplanchnic nerves, as well as the brain and spinal marrow, and see if there can be found anything worth studying.

Symptoms.—As a general rule, this affection sets in suddenly, announcing itself in the physiognomy of the child, changing the expression from pleasing to desponding. He grows fretful, is disturbed with pains in the body. Soon the eyes become sunken, looking dim and dead, with bluish circles around the lower lids. If not motionless, he expresses his sufferings by moans, piercing or pitiful cries. The body is cramped, the skin cold, clammy or dry, shrunken and bluish if touched, leaving the impress of the fingers. The pulse quick and weak; the tongue moist, cold, and covered with a thin mucus through which appear the bright red papillæ.

In the beginning of the attack the child is occasionally seized with vomiting; soon this becomes incessant, the little patient ejecting whatever food or drink is offered him. His thirst is intense. The materials at first vomited are the food or drink which may have been given. This soon changes into a yellow or greenish substance like coffee diluted with milk, or dirty water. The vomiting is succeeded by the flux of the bowels. At first, the discharges are of a natural appearance, but soon change to yellow, like the yolk of an egg, mixed with slime and froth, which, when exposed to the air, will turn green. A little later, they become green as grass, mixed with white and yellow mucus, streaked with blood; dirty, like the water of washed meat; or else altogether bloody. These passages are often preceded by griping pains; they are sometimes passed unnoticed. Tenesmus and prolapsus ani are in some cases the source of great distress to patients, and annoyance to parents.

Sometimes the vomiting and evacuations simultaneously, or separately disappear, and the senses are lost, and, from being restless and agitated, the child becomes calm. This is succeeded by convulsive twitchings of the legs and arms, contortions of the eyes, clenching of the fists, with the thumbs closed within, as forerunner of a false hydrocephalus.

The thermometer, at this juncture, when applied to the skin, even in the hollow of the axilla, descends, proving the diminution of heat on the external surface of the body. The face assumes a leaden hue, and the nails are blue. In this state, all the efforts of a skilful physician may prove unavailing and the child die from exhaustion. But it sometimes happens that the child, being vigorous, survives this attack, and passes for a few hours into a comatose state, having neither vomiting nor passages from the bowels. Whilst in this condition reaction takes place, and upon awakening he has a few irregular passages, vomiting but seldom or not at all. The food, although taken with dislike, is retained.

In a few days he commences to nourish as usual, and he recovers. The food I generally recommend to weaned children is chopped raw meat.

Treatment.—Various have been the remedies for the treatment of this malady. I will enumerate a few:—Castor oil, sulphate of magnesia, rhubarb, ipecac, sulphuric acid, ether, chloroform, sweet spirits of nitre, liquor potassæ, carbonate of soda, lime water, Dover's powders, belladonna, hyoscyamus, hydrocyanic acid, opium, lactucarium, tridace, extract of poppy, mercurials, logwood, kino, catechu, tannin, chalk, krameria, bismuth, nitrate of silver, gallic acid, lead, iron, zinc, bark, &c. Also leeches, flax seed, bran and herb poultices, mustard poultices, and baths, tepid and cold water baths, &c.

For years the above remedies, either alone or combined, have been used in various ways according to the tact and skill of the practitioner, and his success has been baffled by countless difficulties. Even in these days of enlightenment, when medicine is making such rapid progress, and we find in almost every medical periodical some new remedy for this or that malady—strange to relate, in the bowel complaints of children, excepting a slight difference in the combination of drugs, our treatment continues the same as was followed centuries ago. Let us deviate for a while from the routine of castor oil, ipecac, and calomel, and take in hand the so much talked of drug, the bromide of potassium, and see what success we shall have. At the very sound of its name many of my confreres will deny its efficacy. But, gentlemen, if you will kindly listen to me, I shall submit to your judgment one hundred and sixty-three recorded cases of my gathering; and hope you will arrive with me at the conclusion that the bromide of potassium is an invaluable remedy, nay, a specific in summer complaints.

CASES.—I. Casperina Watterau, one year old, born of healthy German parents; nursed by her mother under a very favorable hygienic condition; has six teeth and is healthy. July 15th, 1868, weather very damp and warm; she was suddenly seized with vomiting and free discharges from the bowels. On arriving at the house I found her very much reduced. Eyes sunken, skin and extremities cold, abdomen flat and shrunken, tongue pale, cold, and covered with mucus, pulse hardly perceptible, and impossible to count. I had the child wrapped in warm flannels, gave calomel and opium, brandy and starch injections. On the 16th no improvement. Continued the starch injections, and prescribed chalk mixture and tincture of kino. On 17th, grows worse. Convulsions set in with total loss of senses. I omitted the above remedies, and prescribed twenty minims of tincture of digitalis with one scruple of bromide of potassium in an ounce of water, giving 10 drops every hour. The improvement was immediate. She had no relapse of the above symptoms, became convalescent, and on the 27th was discharged. I was again called to see her on the 2d of August, and found a phlegmonous abscess on the back of the left leg. Poultices were applied, and on the morning of the 4th, I lanced it, and about four ounces of healthy pus came out. In the evening I saw the little patient, and found her very uneasy. The abscess had dried as if it had never suppurated. In the middle of the night convulsions set in as if produced by pyæmia. The bromide of potassium was resumed to check the convulsions, but had no effect, and during the night of the 5th she died.

II. Joseph Frost, six months old; Born of healthy Irish parents; has two teeth; is fed from the bottle; lives in a healthy neighborhood and in well ventilated appartments. July 29th, he is seized with cramps, vomiting, and almost incessant purging. I prescribed a mixture of a half-drachm of bromide of potassium in two ounces of mucilage, giving thirty drops every two hours. After the third dose all alarming symptoms disappeared, and the child recovered. On the 14th of August, he had a relapse, and his mother, having saved what remained of his medicine, gave it to him, and he was again cured.

III. John Sinott, twenty-eight months old. I took charge of him on the 1st of August. For several days he had been suffering from vomiting and purulent discharges from the bowels. I prescribed aromatic syrup of rhubarb with laudanum, but without effect. On the 2d, I gave twenty drops every hour, of a mixture of ten grains of bromide of potassium, in an ounce of mucilage, with twenty minims of tincture of krameria. After a few doses the child slept, and upon awakening asked for bread and butter. Vomiting ceased. The flux of the bowels changed from purulent to yellow; and the twenty-four to thirty passages every twenty-four hours, diminished to six. He became convalescent, and on the 8th was discharged.

IV. J. A. Crigier, twenty months old; has twelve teeth. According to his mother's statement he has never given trouble, eating and drinking whatever was offered him. On the 3d of August he fell from a chair, striking his buttock, apparently receiving no injury. During the night his bowels became loose, accompanied by vomiting of a dirty-water-like substance. In the morning he had convulsions. During the afternoon I found him very comatose; pulse from ninety-five to one hundred; pupils dilated; hemiplega of the right side; alvine discharges, from twelve to fourteen, every twenty-four hours, of a thin fœtid matter. About every six hours convulsions of ten minutes' duration, accompanied by vomiting. I prescribed revulsives to the lower extremities and arms; and every two hours, twenty drops of a mixture of one drachm of bromide of potassium, in an ounce of mucilage. The motion of the bowels and vomiting ceased.

After a few days, as the comatose and hemiplegic condition continued, iodide of potassium brought matters right. On the 1st of Nov., he had another fall, merely affecting his bowels by free motion; the bromide of potassium immediately stopped it.

V. John Balheimer, thirteen months old; has six teeth; is fed by the bottle. On the 15th of August, I saw the child for the first time. He had been suffering for fourteen days from inflammatory dysentery, having passages of a purulent and bloody nature, from twenty to twenty-four, every twenty-four hours. His thirst was intense; he vomited every fluid offered him. The eyes were sunken, pupils dilated, skin corrugated and spotted blue, body cold, tongue red and dry, pulse imperceptible; no urine. In addition to this he had bronchitis with severe cough. I prescribed one scruple of bromide of potassium, in an ounce of mucilage, with half a drachm of tincture of krameria, twenty drops every two hours. The passages decreased. On the sixteenth day of his sickness, and the second under my care, he was able to eat and sleep. His passages were only four in twenty-four hours, and of a healthy appearance. In order to allay the bronchial cough, I prescribed ten drops of fluid extract of poppy, to be taken every two hours. On the 1st of September he was discharged. A few days later he had a relapse, and on account of distance was placed under the care of a homœopathist, and died.

On the 15th an elder brother was brought to me, suffering in a similar manner from dysentery. The disease was immediately checked by the bromide of potassium.

VI. F. Skerret, two years old; has sixteen teeth. He was suddenly seized with purging and vomiting.

6th of August he was brought to me, twenty-four hours after his attack. I found him restless, emaciated, pulse weak, over one hundred and fifty, skin cold, abdomen shrunken, tongue pale and cold; stomach ejecting all fluids, almost in their natural state; from the bowels dirty-water like, odorless passages. Ten grains of bromide of potassium in an ounce of mucilage, ten drops every hour, abate all symptoms, and the next morning he is able to eat and drink as usual, and reported well.

I have had one hundred similar cases, varying in age from three to thirty months, nursing or weaned. I have treated them with the bromide of potassium, and mucilage, and have never found it to fail.

VII. S. Fay, nine months old; has six teeth; nursed by a healthy mother. After fifteen days of diarrhœa, with from fifteen to twenty passages every twenty-four hours, is cured by a mixture of one drachm of the bromide of potassium, in an ounce of mucilage, twenty drops every hour.

I have had fifteen similar cases in every form, having been sick from fifteen to twenty days before coming to me, all treated and cured by the bromide of potassium.

VIII. D. Burk, two months old; nursed by a healthy mother. The child was taken sick with convulsions, vomiting, and diarrhœa. Weather warm and damp. On the 31st of August, a few days after his attack, I found him with his eyes moving in every direction, but without taking any notice; pupils dilated; feet and hands bluish and cold. The pulse could not be found. He was vomiting every ten or fifteen minutes; stools irregular, but very frequent, varying in color, white, green, and yellow.

Twenty drops of a mixture of one ounce of mucilage with ten grains of bromide of potassium, taken every hour, cured this baby, and five others similarly attacked.

IX. J. Murray, five years old. This child, when born, although very healthy, only weighed three pounds and a-quarter. His mother died three months after confinement of hypertrophy of the heart. His father is a sober, healthy Irishman. The child was fed from the bottle. He struggled along through many diseases, having had hydrocephalus, measels, scarlet fever, diphtheria, nephritis, bronchitis, and, once a year, a severe attack of dysentery, commencing with the warm weather, and lasting until fall, which I found very difficult to cure. On the 26th of August he was seized with a profuse flux of the bowels, accompanied by vomiting; and when brought to me he was almost a corpse. I thought this an excellent case to try the efficacy of the bromide of potassium. I gave half a drachm, in an ounce of water, thirty drops every two hours. His father came to my office the following morning without the child. His first salutation was, "My child is better; why did you not give the same medicine last year?" The child continued to improve, and in a few days was perfectly well.

X. J. McEvoy, twelve and a-half years old: a healthy boy attending school. August 13th, having eaten a bellyful of green apples, he was seized with cramps in the belly and legs, colic pains, vomiting, and loose bowels. For forty-eight hours his parents doctored him with castor oil, paregoric, cholera drops, mustard baths, &c., &c., but without giving relief. I was sent for on the 15th, the third day of his sickness. I found him with eyes sunken, skin cold and corrugated, extremities bluish, which upon being touched would leave the white impress of the fingers for more than twenty-five seconds. The abdomen was almost stuck to the vertebral column; breath hurried and hot; tongue pale; voice scarcely audible. The boy was shivering with cold, although covered with mustard plasters, blankets, etc., and in an exceedingly hot, low-ceilinged room on the top floor. His thirst was intense. The drinks given him were, rice-water, beef-tea, brandy and water, ice-water, milk-whey, &c. No sooner taken than ejected indiscriminately, both up and down, almost in their natural state. I thought this case almost hopeless, but trusting to the efficacy of the bromide of potassium, I prescribed a-half drachm in an ounce of water, a teaspoonful every two hours; also a flax-seed meal poultice over the abdomen. The second dose caused great reaction, stopping the vomiting and flux of the bowels, and the boy fell asleep. The next morning I found him in his hot, unhealthy room, laughing and well.

I have had twenty-five similar cases, ranging from twelve to forty-six years of age.

XI. D. Burk, twenty-four years old; sculptor. On the 15th of August, while digging a hole in his courtyard, near the privy, in the heat of the sun, he was seized with vomiting, discharges from the bowels, and cramps. At first the passages were brown and fetid, and the vomit of a yellowish and greenish color. When I saw him the following day the conjunctiva of both eyes was injected; pulse full, over 125 per minute; skin hot and dry; tongue moist, but breath very warm. The vomiting had almost ceased; cramps were continuing in the legs; the passages had changed from brown to the color of meat-washings, copious and very painful. I ordered him to be sponged with cold water and bay rum; his limbs rubbed with a liniment of olive oil and chloroform; and gave internally one drachm of bromide of potassium in two ounces of mucilage, with twenty minims of the tincture of krameria, thirty drops every hour. After the third dose his skin became cool, a genial perspiration diffused itself, and he fell asleep. From 3 P.M. to 11 A.M., he had but three scanty passages, yellowish and without pain. The third day he was well.

I had two more similar cases; one, a plumber, becoming sick after opening a waste-pipe; the other, a carpet weaver, living in Newark, N. J., in a damp low region. When this last-mentioned man came to me, he had been sick for twenty-one days, with clear bloody passages, three every two hours. I prescribed the bromide of potassium as above, and in three days he was able to work.

XII. Mrs. Debaun, twenty-five years old; of nervous temperament; the mother of several children; lives in one of the shanties in 36th street, between 10th and 11th avenues. She is very fretful, crying at trifles. She keeps constantly brooding over the loss of a daughter, and is almost crazy. From fretting, loss of rest, and want of nourishment, she is seized with nervous dysentery of several days' duration. One drachm of the bromide of potassium in an ounce of water, a teaspoonful every two hours, cures her.

XIII. J. McDonald, four years old. For several days he had bloody dysentery and tenesmus with prolapsus ani. After a few doses of the bromide of potassium, the dysentery ceased. Unfortunately, from neglecting to return the rectum, it became strangulated and mortified; and consequently the child died.

XIV. J. Higgins, a healthy infant, deprived of the breast from his mother's dying seven days after confinement. When only three days old he began to suffer from retention of meconium. All nourishment was ejected with great force, even the breast milk of a woman confined five days before his birth. The stomach, from the irritating action of the meconium, could not retain either medicine or food, nor could the bowels be moved. Ten grains of bromide of potassium in an ounce of orange-flower water, ten drops every hour, put a stop to all disagreeable symptoms; the baby slept, its bowels moved, and after the discharge of the meconium, it commenced to nourish from the bottle without any further disagreeable results.

I had three similar cases, not owing to the mother's death but the infants being deprived of the maternal nourishment from other causes.

XV. Mr. A. B——, seventy-five years old; of nervous temperament; healthy, but weak from advanced age. In the beginning of September he went to Tarry-town to spend a few days with friends. After taking a ride in an open wagon, along the banks of the Hudson, the atmosphere being raw and damp, he found himself, upon returning home, stiff and unable to walk. He was brought to New York, and on the 7th of September I was called to see him. I found hemiplegia of the left side, and commenced to treat him with the usual remedies for this affection. Everything progressed favorably until the 16th, when the life of my patient was threatened by a profuse painless diarrhœa. I used various remedies to stop this, but without success. On the 20th, the fourth day of the attack, a teaspoonful of a mixture of two ounces of mucilage of quince seed and one drachm of bromide of potassium, taken every two hours, saved his life.

XVI. Joseph O'Brien, twenty-one months old; has sixteen teeth. He was taken sick in the beginning of September, with diarrhœa and vomiting, and continued in this state until the 1st of October, when I was sent for. I found the child emaciated; pulse very high and feeble; incessant vomiting, and frequent putrid passages from the bowels. Abdomen very tender, apparently from great sensitiveness of the mesenteric glands. As an experiment, I used the bromide of potassium, and succeeded in arresting the vomiting and flux of the bowels. But on the 8th the patient died of mesenteritis.

XVII. After a good many days of extreme fatigue, loss of rest, want of proper diet, and abuse of iced water, whilst in a state of perspiration, I was myself seized, August 24th, with painless diarrhœa, lasting for two days, having from fifteen to twenty passages in twenty-four hours. Although the weather was excessively warm, I was obliged to clothe myself with heavy winter flannels. My abdomen, particularly, was very cold. Internally my breast was burning; externally, chilly. Pulse one hundred, but weak; tongue cold; throat hot and constricted. My head was as if in a furnace, although otherwise without pain. Urine scant, dribbing a few drops when I went to stool. At every evacuation I felt as if my intestines were sinking from me. I was restless and shaky, as if seized with palsy. During the evening of the 26th, I took, every hour, five-grain doses of bromide of potassium in pure water. No sooner was it taken, than a pleasing sensation of heat diffused itself through the abdomen, and my head became cool. The creeping chills were replaced by a gentle heat. After the second dose I fell asleep for six hours, perspiring profusely; and in the morning called for my usual cup of coffee and ice, and resumed my practice.

XVIII. During the riot in this city, a boy about twelve years old was struck between his shoulders with a club, by one of the rioters. He fell insensible, and was carried home. A few minutes after his arrival he commenced to vomit the contents of the stomach; afterwards a dirty and yellow watery substance. For two days nothing could stop the vomiting. On the third day I gave him the bromide of potassium, not with the intention of stopping the vomiting, but to make him sleep. No sooner was the first dose administered than the vomiting ceased, and he slept.

XIX. J. McG——, thirty-four years old; porter in a store in Chambers street. Some time in April, 1866, after pumping water, and whilst in a state of perspiration, he assisted the plumber to open a waste-pipe, from which arose a stench so terrible as to cause all hands to vacate the premises. He remained to finish his work, but was soon obliged to stop, being suddenly seized with vomiting. He was conveyed to his home in 28th street, between 6th and 7th avenues. He sent for his family physician, and after five weeks of unsuccessful medication was given up. The poor man, in an exhausted state, sent for me. I found him very weak; pulse ranging from one hundred and twenty to one hundred and thirty, small and compressible; vomiting every particle of food taken, whether solid or liquid. I used various remedies, but after five weeks of medication I was as unsuccessful as my predecessor. Fearing for the life of my patient, I called in consultation Professor A. Clarke. After a careful examination of the case he arrived at the same conclusion which I had reached, namely, that the man was affected with miasmatic nervous fever producing vomiting, and that a fair trial of the sulphite of soda, which I had already given, would probably insure success. I continued the medicine for forty-eight hours, but was disappointed. Every conceivable remedy was used without the desired results. The patient growing weaker and restless, I prescribed bromide of potassium to cause sleep. He slept, and, to my astonishment, the vomiting ceased. As he was taking nitrate of silver, bismuth, opium, and belladonna, I attributed the relief to these drugs, and not to the bromide of potassium, and so let it pass unnoticed.

XX. During the summer of the same year, a child of about eighteen months was taken sick with diarrhœa and vomiting. One remedy after another was used without giving relief. The child being restless both night and day, and troubled with strabismus, I gave him bromide of potassium to make him sleep. His bowels ceased moving, and his eyes returned to their normal state.

Notwithstanding these successes, my attention was not directed to the remedy until the first case spoken of in this paper, when I used the bromide of potassium, not as a remedy for the disease, but to relieve the child from convulsions. When I saw it arresting vomiting and the flux of the bowels, I concluded it must be a good remedy for summer complaint.

Now let us speak on the merits of the drug. Taking for granted, that in matters of fact, in medicine particularly, we can judge better *a posteriori* than *a priori*, I submit my cases and conclusions to this enlightened body. Not accepting the doctrine of some of the modern writers, that the intestinal flux is a symptom of some inflammatory affection; but adopting the opinion of the celebrated Capuron, that infancy being naturally the age of nervous sensitiveness, susceptible to internal and external impressions, easily irritated or calmed, and judging from several other cases besides those just read, I think that in its beginning, summer complaint arises from an over-excitement of the nervous and vascular systems, and that therefore the bromide of potassium affects it, and acts as a sure cure.

I cannot better explain the action of the bromide of potassium on the system, than by quoting the words of Headland, found on page 272 of his work, "*On the Action of Medicines:*" "The bromide of potasium, a medicine of the mineral kingdom, has been much recommended lately for its power of producing sleep; it is, however, not a true soporific, but rather a general sedative. As far as my experience goes, it acts by allaying irritability of the brain, spinal cord, and sexual system, and thus may indirectly cause sleep." And on page 283,—"The bromide of potassium, a remedy of recent introduction, is exceptional among nerve medicines, as belonging to the mineral instead of the vegetable kingdom. It quiets the nervous system generally, allays pain, promotes sleep, and subdues a morbid irritability of cutaneous or mucous surfaces, by its influence over functional disorders of the nervous centres. It is only indirectly soporific, as far at least as I have been able to judge." Mr. Gubler considers the bromide of potassium a general sedative to the nervous system, allaying irritability of the mucous membranes, and at the fauces and genital passages especially.

He thinks it hypnotic, causing sleep by its sedative action on the whole nervous system. M. Vigouroux thinks it acts by diminishing vascularity of the great nervous centre. It is therefore the remedy, par excellence, for the nervous complaints that are common in large cities.—(*L' Union Medicale, 1864.*)

I have never discovered any unpleasant effects produced by the use of the bromide of potassium, and have always found it to answer my purposes. If locally applied, it reduces the excessive heat found in the mouth of the baby, caused by the inflamed condition of the gums. On introducing the thermometer into the mouth and anus of the baby, I have seen it fall four or five degrees. The drug is an anæsthetic to the nerves of the mucous membrane of the alimentary canal from the mouth to the rectum, of the urethra, the conjunctiva and the nares. It is also a diuretic. In cases where the urine has not been voided for twenty-four hours, after the first dose of the bromide it is freely passed; the skin commences to feel warm and becomes covered with a gentle perspiration, removing uræmic spmptoms, causing general reaction, and invariably the decrease of the intestinal flux, and an absolute cessation of vomiting.

The medicine being almost tasteless, and of no bulk, it is easily taken by the most fastidious and troublesome child. I generally prescribe from ten to thirty grains in an ounce of vehicle, either mucilage or orange-flower water for their pleasant taste; the dose being ten to thirty drops every hour or two, varying according to the age of the patient, and the acuteness of the case. I seldom use astringents with it; but if required I select the tincture of krameria, as less disgusting than kino and others. For local application I mix the salt with mel rosarum, generally using one scruple of the first to an ounce of the latter, permitting the mother to rub it on the guns with her finger *ad libitum.* When used in large doses, I have not found very satisfactory results from the bromide of potassium; but I always succeeded with minute doses.

You will observe that of the cases I have just reported, only three died, and the fatal result in these was due not to the bowel complaint, but to other causes. I had also four cases which I purposely and obstinately treated according to the generally adopted system. To my regret I must confess, that but one, after a long and severe struggle, recovered; the other three died.—*New York Medical Record.*

